# A cost-effectiveness analysis of the combination of serplulimab with chemotherapy for advanced esophageal squamous cell carcinoma: insights from the ASTRUM-007 trial

**DOI:** 10.1186/s12962-024-00516-5

**Published:** 2024-01-27

**Authors:** Jiahui Li, Chaoqun Xu, Suyun Yuan

**Affiliations:** Department of Radiation Oncology, Kexin Cancer Hospital, Changsha, 410000 China

**Keywords:** Cost-effectiveness analysis, Serplulimab, Chemotherapy, Advanced esophageal squamous cell carcinoma, ASTRUM-007 trial

## Abstract

**Background:**

Combined serplulimab and chemotherapy demonstrated improved clinical survival outcomes in patients with advanced esophageal squamous cell carcinoma (ESCC) and PD-L1 combined positive scores (CPS) ≥ 1. The present study aimed to evaluate the economic viability of integrating serplulimab in combination with chemotherapy as a potential therapeutic approach for treating ESCC in China.

**Methods:**

A Markov model was constructed to evaluate the economic and health-related implications of combining serplulimab with chemotherapy. With the incremental cost-effectiveness ratio (ICER), costs and results in terms of health were estimated. For assessing parameter uncertainty, one-way and probabilistic sensitivity studies were carried out.

**Results:**

The combination of serplulimab and chemotherapy yielded incremental costs and QALYs of $3,163 and 0.14, $2,418 and 0.10, and $3,849 and 0.15, respectively, for the overall population as well as patients with PD-L1 CPS1-10 and PD-L1 CPS ≥ 10. This corresponds to ICER values per QALY of $23,657, $23,982, and $25,134. At the prespecified WTP limit, the probabilities of serplulimab with chemotherapy being the preferred intervention option were 74.4%, 61.3%, and 78.1% for the entire patient population, those with PD-L1 1 ≤ CPS < 10, and those with PD-L1 CPS ≥ 10, respectively. The stability of the presented model was confirmed through sensitivity studies.

**Conclusions:**

In conclusion, the combination of Serplulimab and chemotherapy showed excellent cost-effectiveness compared to chemotherapy alone in treating PD-L1-positive patients with ESCC in China.

**Supplementary Information:**

The online version contains supplementary material available at 10.1186/s12962-024-00516-5.

## Introduction

Esophageal cancer (EC) is the seventh most common and the sixth deadliest cancer worldwide, with 604,100 diagnosed cases and 544,076 deaths yearly [1]. In 2022, it was estimated that there would be approximately 346,633 diagnosed EC cases and 323,600 EC-related deaths in China [2]. EC cases are classified histologically into two subtypes, namely, the predominant esophageal squamous cell carcinoma (ESCC), comprising 84% of cases, and the less common esophageal adenocarcinoma (EAC) [3, 4]. EC typically exhibits a poor prognosis, with more than 50% of patients presenting with unresectable disease or distant metastases at diagnosis. Consequently, the 5-year survival rate for EC is significantly low at 18% [5].

Despite development in combination chemotherapy regimens, such as CF (5-fluorouracil with cisplatin) and TP (paclitaxel with cisplatin), which are currently the conventional first-line treatments for patients with advanced ESCC (aESCC), overall results for these patients remain unsatisfactory. Clinical trials have reported poor median overall survival (mOS) rates of 5.5 between 10 months, as well as progression-free survival (mPFS) rates of 3.6-7 months [6, 7]. The introduction of immune checkpoint inhibitor (ICI) therapy has revolutionized the standard approach for treating aESCC patients, substantially enhancing chemotherapy efficacy and achieving a mOS of 10 to 12 months [8–12]. Nonetheless, there is an urgent need to discover novel anticancer medicines and therapy techniques to address aESCC effectively. Furthermore, current therapy regimens demand regular improvements.

Serplulimab, or HLX10, is a fully-humanized monoclonal IgG4 antibody explicitly targeting the programmed cell death protein 1 (PD-1) (Shanghai Fuhong Hanlin Biopharmaceutical Co., LTD). The compound has exhibited significant anticancer properties and is safe for treating several forms of cancer [13–15]. Recent ASTRUM-007 (NCT03958890) trial findings have revealed significant benefits of first-line immunotherapy plus CF chemotherapy in treating aESCC patients. mOS and mPFS have significantly improved as a result of this combination therapy in a variety of patient populations: overall patients (mOS: 15.3 vs. 11.8 months; hazard ratio [HR]: 0.68; 95% confidence interval [CI]: 0.53–0.87; *P* = 0.0020; mPFS: 5.8 vs. 5.3; HR: 0.60; 95% CI: 0.48–0.75; *P* < 0.0001), patients carrying a PD-L1 combined positive score (CPS) ranging between 1 and 10 (mOS: 14.2 vs. 11.4; HR: 0.74; 95% CI: 0.54–1.03; *P* = 0.066; mPFS: 5.7 vs. 5.3; HR: 0.70; 95% CI: 0.52–0.94; *P* = 0.017), and patients carrying a PD-L1 CPS ≥ 10 (mOS: 18.6 vs. 13.9; HR: 0.59; 95% CI: 0.40–0.88; *P* = 0.0082; mPFS: 7.1 vs. 5.3; HR: 0.48; 95% CI: 0.34–0.68; *P* < 0.0001). The National Medical Products Administration (NMPA) authorized the New Drug Application (NDA) for first-line serplulimab with chemotherapy for treating ESCC due to the treatment’s potential clinicopathological benefits and excellent safety profile. Furthermore, the Chinese Guidelines for Radiotherapy of Esophageal Cancer in 2022 have also recommended this interventional protocol [16, 17].

Serplulimab has demonstrated efficacy and safety. Nevertheless, few assessment has been conducted thus far regarding the cost-effectiveness of serplulimab concerning patients with ESCC. Considering the increasing scope of treatment applications arising from advancements in immunotherapy, it is crucial to evaluate the financial feasibility of these therapies. Assessing the cost-effectiveness of these immunotherapeutic regimens can offer vital information to policymakers, healthcare providers, and patients concerning the evidence-based value of adopting new therapeutic interventions. Previous research has found that serplulimab is not cost-effective compared with chemotherapy as a first-line treatment for ESCC patients. However, after careful examination, it was found that these several studies were considering the charitable donation of drugs or preferential policies of the company, and could not fully show the real-world research results [19, 20]. In the current study, we investigated the financial viability of serplulimab plus chemotherapy as the initial treatment for PD-L1-positive aESCC patients in China.

## Materials and methods

The Consolidated Health Economic Evaluation Reporting Standards (CHEERS) criteria for economic evaluation established by the International Society for Pharmacoeconomics and Outcomes Research were used to guide this analysis [18] (Supplementary Materials Table S1).

### Population and intervention

Our model included a population similar to the ASTRUM-007 clinical trial, comprising treatment-naive patients with locally advanced or metastatic ESCC and a PD-L1 CPS ≥ 1 [21]. In this economic evaluation, eligible participants comprised individuals aged 18–75 years presenting with previously untreated, histologically confirmed, inoperable locally advanced or metastatic esophageal squamous cell carcinoma (ESCC) characterized by PD-L1 positivity (combined positive score [CPS] ≥ 1). Inclusion criteria involved the presence of at least one measurable lesion determined through central imaging in accordance with Response Evaluation Criteria in Solid Tumors version 1.1 (RECIST v1.1), adequate organ function, and Eastern Cooperative Oncology Group (ECOG) performance status of 0–1. Central testing for PD-L1 immunohistochemistry was conducted on tumors. Exclusion criteria encompassed prior exposure to PD-1 or PD-L1 inhibitors, the existence of central nervous system metastases, or the presence of active infection or autoimmune diseases. In all, 511 patients were randomized in a 2:1 ratio for serplulimab (*n* = 368) or placebo (*n* = 183) treatment, along with chemotherapy (cisplatin with 5-fluorouracil [CF]). Of the patients who received serplulimab plus CF, 206 (56%) exhibited PD-L1 expression levels ranging from 1 to CPS < 10, while 162 (44%) had PD-L1 levels of ≥ 10. In the placebo with CF cohort, 79 (43%) had PD-L1 levels of ≥ 10 [21]. In the ASTRUM-007 trial, patients were administered either serplulimab or placebo at 3 mg/kg per day over up to 2 years, together with cisplatin at 50 mg/m^2^ per day for up to 8 cycles, and 5-fluorouracil at 1,200 mg/m^2^ on days 1 and 2 for a maximum of 12 cycles. Cisplatin and 5-fluorouracil were administered intravenously every two weeks [21]. The patients’ average age, body weight, and body surface area were 64 years, 65 kg, and 1.72 m^2^, respectively [19–21] (Table 1).

**Table 1:** Model parameters: key clinical and health preference data

Parameters	Baseline value	Range		Reference	Distribution
		Minimum	Maximum		
**Clinical data**					
**Weibull survival model for OS of serplulimab plus CF**					
Overall patients	Scale = 0.02617, Shape = 1.16846	-	-	(19)	-
Patients with PD-L1 expression level of 1 ≤ CPS < 10	Scale = 0.029888, Shape = 1.176584	-	-		-
Patients with PD-L1 CPS ≥ 10	Scale = 0.016952, Shape = 1.254789	-	-		-
**Weibull survival model for PFS of serplulimab plus CF**					
Overall patients	Scale = 0.10927, Shape = 0.97361	-	-	(19)	-
Patients with PD-L1 expression level of 1 ≤ CPS < 10	Scale = 0.10943, Shape = 1.05893	-	-		-
Patients with PD-L1 CPS ≥ 10	Scale = 0.087778, Shape = 0.989156	-	-		-
**Weibull survival model for OS of placebo plus CF**					
Overall patients	Scale = 0.020542, Shape = 1.404457	-	-	(19)	-
Patients with PD-L1 expression level of 1 ≤ CPS < 10	Scale = 0.0127079, Shape = 1.6243877	-	-		-
Patients with PD-L1 CPS ≥ 10	Scale = 0.030741, Shape = 1.212235	-	-		-
**Weibull survival model for PFS of placebo plus CF**					
Overall patients	Scale = 0.0931, Shape = 1.32774	-	-	(19)	-
Patients with PD-L1 expression level of 1 ≤ CPS < 10	Scale = 0.06808, Shape = 1.50072	-	-		-
Patients with PD-L1 CPS ≥ 10	Scale = 0.06808, Shape = 1.524542	-	-		-
**Risk for main AEs in serplulimab plus CF group**					
Anemia	0.180	0.144	0.216	(19)	Beta
Hyponatremia	0.050	0.040	0.060	(19)	Beta
Neutrophil count decreased	0.190	0.152	0.228	(19)	Beta
White blood cell count decreased	0.110	0.088	0.132	(19)	Beta
**Risk for main AEs in placebo plus CF group**					
Anemia	0.200	0.160	0.240	(19)	Beta
Neutrophil count decreased	0.170	0.136	0.204	(19)	Beta
White blood cell count decreased	0.070	0.056	0.084	(19)	Beta
**Proportion of receiving active second-line treatment**					
Serplulimab plus CF	0.520	0.416	0.624	(19)	Beta
CF	0.380	0.304	0.456	(19)	Beta
**Utility and disutility**					
Utility of PFS	0.680	0.544	0.816	(21)	Beta
Utility of PD	0.420	0.336	0.504	(21)	Beta
Disutility of anemia	0.074	0.059	0.089	(21)	Beta
Disutility of neutrophil count decreased	0.090	0.072	0.108	(21)	Beta
Disutility of white blood cell count decreased	0.090	0.072	0.108	(21)	Beta
Disutility of hyponatremia	0	-	-	(21)	-
**Body weight (kilogram)**	65	52	78	(19, 20)	Normal
**Body surface area (meters** ^**2**^ **)**	1.72	1.38	2.06	(19, 20)	Normal
**Discount rate**	0.05	0	0.08	(21, 24)	Uniform

*Abbreviation*: OS, overall survival; CF, cisplatin and 5-fluorouracil; PD-L1, programmed death ligand 1; CPS, combined positive score; PFS, progression-free survival; PD, progressive disease; AEs, adverse events

In situations of progressing disease (PD) or unacceptable adverse effects (AEs) during first-line medication, 139 (38%) patients in the serplulimab with CF group and 95 (52%) individuals in the placebo with CF cohort received second-line therapy [19] (Table 1). Second-line treatment followed the National Comprehensive Cancer Network (NCCN) and Chinese Society of Clinical Oncology (CSCO) criteria, with paclitaxel as the chosen intervention. Other patients received the best supportive care (BSC) [24, 25]. Terminal care services were provided to elderly patients in the advanced stages of their lives. The comprehensive medication schedule is summarized in Table S2 in the supplementary materials.

### Model construction

The decision-analytical Markov model incorporated three distinct and mutually exclusive states to simulate the overall progression of ESCC: PFS, progressive disease (PD), and mortality outcomes (refer to Supplementary Materials Figure S1). At the commencement of the trial, all participants were in the progression-free survival (PFS) state of the Markov model and exhibited the possibility of transitioning to either the PD or death states. During subsequent cycles, PD patients either remained in the PD cohort or transitioned to the deceased cohort, signifying the cessation of circulation for the individual. The operational framework of the model consisted of recurring 2-week cycles, including a 10-year temporal scope for study. Within this timeframe, it was observed that mortality was encountered by almost 99% of the patients. The TreeAge Pro v.2021 (https://www.treeage.com) platform was utilized to develop the model.

The primary outcomes were the total average expenditure, incremental cost-effectiveness ratio (ICER) values, life-years (LYs), and quality-adjusted LYs (QALYs). Expenditure and survival estimations utilized a 5% annual discount rate [22, 23]. ICER values were computed by dividing incremental costs by incremental increases in QALYs and then compared to a WTP cutoff of $36,438/QALY, representing three times China’s gross domestic product [26].

### Model of survival and transitional probabilities

We conducted parameter distribution fitting on the survival curve to achieve long-term patient survival information well beyond the typical follow-up duration in clinical trials. We simulated a population resembling the characteristics of patients in the ASTRUM-007 trial and extracted short-term survival information from OS and PFS Kaplan-Meier curves using GetData Graph Digitizer (version 2.26, available at: http://www.getdata-graph-digitizer.com/index.php). The choice of exponential, log-logistic, log-normal, Gompertz, and Weibull distributions for fitting survival curves was determined by a combination of clinical reasoning, visual examination, and the application of Bayesian and Akaike information criteria (BIC and AIC) [27] (Supplementary Materials Figure S2 and Table S3).

The Weibull distribution performed the best at reconstructing the data for each patient. Each cycle’s time-dependent transitional probabilities were computed as follows: (1 - exp {𝜆(t-u)^𝛾^– 𝜆𝑡^𝛾^}), where u is the Markov cycle, t denotes the present model cycle, λ and γ refer to the scale and shape parameter, respectively. These parameters were calculated with R (version 4.1.1, available at: http://www.rproject.org) [27, 28]. Refer to Table 1 for more information on the estimated model parameters.

### Utility and cost input

A health utility preference between 0 and 1 was designated to individual Markov health conditions, with 0 denoting demises and 1 referring to perfect health. Because data on quality of life (QoL) from the ASTRUM-007 trial was unavailable, utility values were calculated from earlier studies on aESCC. The average utility score allocated to patients within the condition of progression-free survival (PFS) was 0.68, whereas individuals in the progressive disease (PD) condition exhibited a reduced utility score of 0.42 [28]. Adjusting average utility values to account for the disutility associated with treatment-related adverse events was also examined [29].

We only considered direct medical care-related expenses, such as drug expenditure, treatment costs for AEs, regular tumor imaging, laboratory testing, PD-L1 testing, administration, BSC, and terminal care (Table 2). Prices for drugs and PD-L1 tests were sourced from real-world hospitals, while other direct medical expenses were derived from published reports [28–31]. Furthermore, this study only considered expenses linked to grade ≥ 3 treatment-related AEs with an incidence of ≥ 5%, including anemia, reduced white blood cell count, diminished neutrophil count, and hyponatremia [26–28]. The AEs cost was calculated by multiplying the treatment cost associated with each AE event by its corresponding incidence rate. Prices were reported in US dollars, using a conversion rate of *$1 =¥7.0559* (May 2023).

**Table 2:** Cost estimates ($)

Parameters	Baseline value	Range		Reference	Distribution
		Minimum	Maximum		
**Drug cost, $/per cycle**					
Serplulimab	198	158	238	Real-world	Gamma
Cisplatin	12	10	14	Real-world	Gamma
5-fluorouracil	127	102	152	Real-world	Gamma
Paclitaxel	180	144	216	Real-world	Gamma
**Cost of AEs**					
Serplulimab plus CF	65	52	78	(21, 28, 29)	Gamma
CF	60	48	72	(21, 28, 29)	Gamma
**Laboratory per cycle**	53	42	64	(21)	Gamma
**Tumor imaging per cycle**	162	130	194	(21)	Gamma
**Administration per cycle**	12	10	14	(21)	Gamma
**PD-L1 test per patient**	86	69	103	Real-world	Gamma
**Best supportive care per cycle**	70	56	84	(21)	Gamma
**Terminal care per patient**	1,402	1,122	1,682	(27)	Gamma

*Abbreviation*: AEs, adverse events; CF, cisplatin and 5-fluorouracil; PD-L1, programmed death ligand 1

### Sensitivity analyses

We conducted a sequence of sensitivity analyses to assess the potency of the decision analytical model and investigate the impact of uncertainty in individual variable values on the overall model outcomes. To perform one-way sensitivity studies, the parameters were categorized within a range of ± 20% relative to the baseline values, per the standard approach for evaluating the impact of uncertainty on ICERs [32]. A probabilistic sensitivity analysis was conducted by executing 10,000 Monte Carlo simulations. In each simulation, the parameters were sampled randomly from their recommended distributions, taking into account the parameter type [33]. As recommended, we employed gamma distributions for costs and beta distributions for both adverse event (AE) incidence and utility values [32, 34]. To illustrate the outcomes of these sensitivity analyses, tornado diagrams, acceptability curves, and scatter plots were utilized.

Additionally, we evaluated the cost-effectiveness outcomes for various subgroups using forest plots obtained from the ASTRUM-007 trial [21]. Subgroup stratification was based on age, sex, Eastern Cooperative Oncology Group (ECOG) performance, disease status, and smoking status. Except for the HRs for OS and PFS, the remaining subgroups in the study utilized the same data due to limited data accessibility.

## Results

### Base-case analysis

In patients with aESCC, introducing serplulimab in conjunction with CF led to a notable increase of 0.14 QALYs or 0.27 LYs. However, this improvement in patient outcomes came at an additional cost of $3,163 compared to using CF alone. In contrast, applying TreeAge Pro 2021 in model development resulted in an ICER of $23,657 per QALY ($11,975 per life-year). Regarding patients with PD-L1 levels ranging from 1 to less than 10 (CPS 1 ≤ CPS < 10) and PD-L1 CPS ≥ 10, the addition of serplulimab to CF was projected to result in an extra 0.10 overall QALYs (0.21 overall LYs) and 0.15 overall QALYs (0.29 overall LYs), incurring higher costs of $2,418 and $3,849 compared to CF, respectively. This corresponded to ICERs of $23,982 per QALY ($11,367 per LY) and $25,134 per QALY ($13,672 per LY) (Table 3).

**Table 3:** Results of the base-case analysis

Treatment	Total cost, $	Overall LYs	Overall QALYs	ICER, $	
				per LY	per QALY
**Overall population**					
Serplulimab plus CF	7,241	0.87	0.44	11,975	23,657
CF	4,078	0.60	0.30	-	-
**Patients with PD-L1 expression level of 1 ≤ CPS < 10**					
Serplulimab plus CF	6,333	0.76	0.38	11,367	23,982
CF	3,915	0.55	0.28	-	-
**Patients with PD-L1 CPS ≥ 10**					
Serplulimab plus CF	8,286	0.97	0.49	13,672	25,134
CF	4,437	0.68	0.34	-	-

*Abbreviation*: LYs, life-years; QALYs, quality-adjusted life-years; ICER, incremental cost-effectiveness ratio; CF, cisplatin and 5-fluorouracil; PD-L1, programmed death ligand 1; CPS, combined positive score

### Sensitivity analysis

**Fig. 1:**
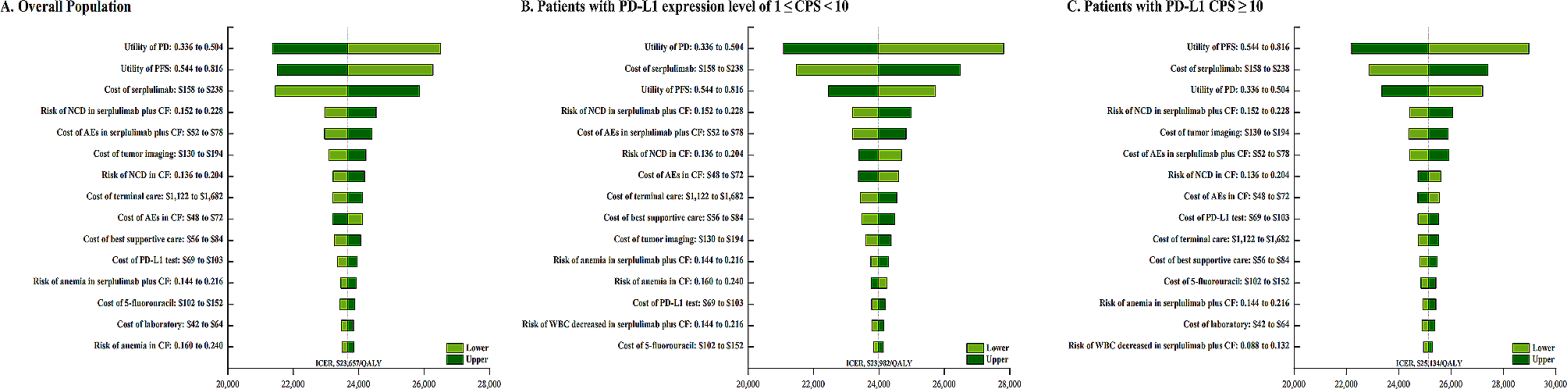
The One-way Sensitivity Analyses for Serplulimab plus CF Strategy Compared to Placebo plus CF Strategy in the Overall Patients (A), Patients with PD-L1 Expression Level of 1 ≤ CPS < 10 (B), Patients with PD-L1 CPS ≥ 10 (C). Abbreviation: PFS, progression-free survival; PD, progressive disease; CF, cisplatin and 5-fluorouracil; PD-L1, programmed death ligand 1; NCD, Neutrophil count decreased; WBC, White blood cell; AEs, adverse events; QALY, quality-adjusted life-year

**Fig. 2:**
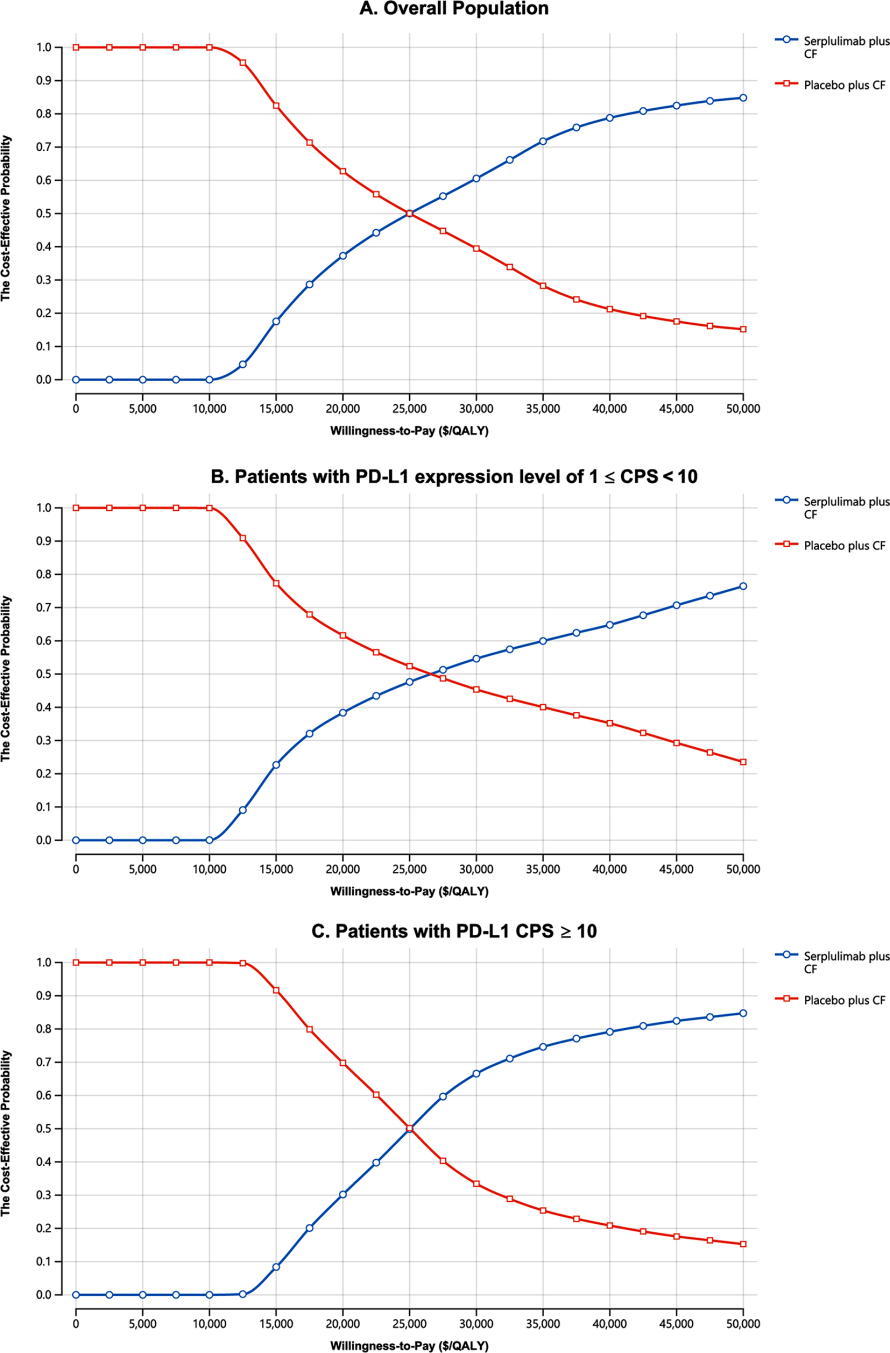
The Cost-effectiveness Acceptability Curves for Serplulimab plus CF Strategy Compared to Placebo plus CF Strategy in the Overall Patients (**A**), Patients with PD-L1 Expression Level of 1 ≤ CPS < 10 (**B**), Patients with PD-L1 CPS ≥ 10 (**C**). *Abbreviation*: QALY, quality-adjusted life-year; CF, cisplatin and 5-fluorouracil

The tornado chart obtained by a one-way sensitivity analysis shows that the utility values for PD and PFS substantially influenced the model. Variations in these values, ranging from 0.336 to 0.816, corresponded to ICERs ranging from $21,081 per QALY to $28,971 per QALY. Additionally, the cost of serplulimab, the risk of decreased neutrophil count in serplulimab plus CF, and the cost of AEs also significantly impacted the study outcomes (Fig. 1). In comparison, the model’s results were minimally affected by factors such as the cost of chemotherapy, the negative impact on well-being associated with treatment-related AEs, and the expense of second-line treatment (Fig. 1). The expenditure feasibility acceptability curve from the probabilistic sensitivity analysis showed an expanding possibility of serplulimab with CF being regarded as a practical expenditure as the willingness to pay (WTP) cutoff point increased (Fig. 2). As shown in the scatter plots (Supplementary Materials Figure S3), at a WTP cutoff of $36,438 per QALY, the expenditure feasibility probabilities for serplulimab with CF treatment in the three patient groups were 74.4%, 61.3%, and 78.1%, respectively.

The findings of the subgroup analysis indicated that the combination of serplulimab and CF consistently demonstrated cost-effectiveness as a viable therapy choice specifically for Chinese patients. The ICER was significantly reduced in patients with an ECOG performance of 0, resulting in an ICER of $19,706 per QALY and a 77.3% probability of cost-effectiveness. Subsequently, current or former smokers and patients aged < 65 years also exhibited favorable ICER values (Supplementary Materials Table S4).

## Discussion

Serplulimab combined with chemotherapy is a novel therapeutic approach for patients with aESCC and PD-L1 positivity, according to the results of the prospective phase 3 ASTRUM-007 randomized clinical trial, challenging the traditional classification and treatment paradigm of ESCC. In China, the total annual direct medical costs related to EC are projected to show a substantial 128.7% growth from 2013 to 2030, escalating from $33.4 billion to $76.4 billion [35]. The EC incidence is projected to rise from 61.00 per 100,000 to 64.5 per 100,000, reaching its highest point at 67.9 per 100,000 in 2020. Furthermore, up to 40% of ESCC patients are characterized by PD-L1 overexpression [35, 36]. Rising healthcare expenses highlight the importance of value-based oncology. Considering the high demand for treating aESCC with PD-L1 overexpression and an increasing interest in evaluating therapeutic options economically, this study undertook a detailed financial analysis of serplulimab use in specific clinical settings.

From the standpoint of a Chinese payer, this represents one of the initial assessment of the expenditure practicality of serplulimab combined with chemotherapy against placebo + chemotherapy across three diverse PD-L1 CPS groups. The Markov model analysis yielded ICER estimates of $23,657, $23,982, and $25,134 per QALY for serplulimab plus CF, patients with PD-L1 levels ranging from 1 to less than 10 (CPS 1 ≤ CPS < 10), and those with a PD-L1 CPS ≥ 10, respectively, compared with patients who received placebo plus chemotherapy. Given current drug prices, these values were significantly below the WPT threshold, suggesting that these treatments may be cost-effective in China.

Additionally, based on our probabilistic sensitivity analyses, there was a high likelihood of cost-effectiveness for serplulimab plus chemotherapy treatment in these three patient populations in China, with probabilities of 74.4%, 61.3%, and 78.1%, respectively. In patients with lower HRs for OS and PFS, the ICI-based regimen, especially serplulimab plus chemotherapy, produced more positive economic outcomes than placebo plus chemotherapy. In patients who had higher HRs, however, it resulted in less positive economic consequences. This aligns with the evaluation based on stratified PD-L1 expression, demonstrating that the economic outcomes of serplulimab plus chemotherapy treatment are superior in patients with PD-L1 CPS values ≥ 10 compared to those with PD-L1 levels of 1 ≤ CPS < 10, likely due to the former group exhibiting better relative efficacy in response to serplulimab plus chemotherapy treatment. This is consistent with several previous research studies investigating the cost-effectiveness of ICI-based therapies in solid tumors [37–41].

Two recent studies on the cost-effectiveness of sintilimab with chemotherapy for advanced EC indicated that this treatment regimen was more cost-effective for patients with PD-L1 CPS ≥ 10 than those with PD-L1 CPS ≥ 1 or 5 [37, 38]. Two additional articles investigated the expenditure feasibility of ICI-based treatments, specifically pembrolizumab or nivolumab, for non-small cell lung cancer (NSCLC), revealing that these interventions were more cost-effective among PD-L1 ≥ 50% patients than PD-L1 ≥ 1% or 20% patients [39, 40]. As a result, PD-L1 is a poor predictor of superior therapeutic response to PD-L1 or PD-1 inhibitors in several tumor types, including ESCC [42]. Depending on the PD-L1 expression status, these innovative combination treatment regimens should be considered viable choices for ESCC patients, emphasizing the importance of early discovery of these predictive biomarkers to achieve optimal patient outcomes.

The results of the one-way sensitivity analysis showed that changes in individual model parameters did not affect the study findings, confirming the model’s stability. The most important factors influencing the model’s results were the utility values and Serplulimab’s pricing. According to our model results, the cost of serplulimab significantly influenced the ICERs. However, these conditions could be ameliorated through charitable drug donations. Later analysis showed that the cost-effectiveness of the serplulimab and chemotherapy combination would no longer meet the standard for being deemed economically feasible if the price of serplulimab rose by 2.16 times (~ $428 per cycle). Secondly, utility values are multiplied by the time spent in a particular health state to calculate QALYs. Therefore, variations in utility values directly affect the calculation of QALYs, which, in turn, influences the ICER. Moreover, the acceptability curve demonstrated that variations influenced the ICER in the WTP value, which, in turn, was influenced by the per capita GDP of China. In this study, the WTP threshold, based on previous research, was described as 3-fold of the GDP per capita [43].

Given the extensive geographical expanse and rich abundance of resources within China, however, substantial variations in GDP per capita exist among different regions. For instance, the corresponding WTP values per QALY for Beijing, Shanghai, Guangdong, Hubei, Hunan, Guangxi, and Gansu are $80,822, $76,277, $43,281, $39,188, $36,650, $22,200, and $19,127, respectively [35]. Interestingly, in more developed regions of China, serplulimab plus chemotherapy may be the best intervention strategy for ESCC patients, although this may not be the case in less developed areas. Therefore, it is essential to take into account the unique economic circumstances of various regions before authorizing new drugs or therapies in China.

Our subgroup analysis revealed that serplulimab with chemotherapy was potentially practical in expenditure, particularly among individuals with an ECOG performance of 0, followed by current or former smokers and patients < 65 years old who had PD-L1-positive aESCC. Therefore, comparing and validating optimal strategies for specific populations in future studies is essential. When determining whether a new method is acceptable for the target population, Medicare and insurance decisions consider economic aspects in addition to health benefits and patient outcomes. Our cost-effectiveness analysis can provide statistical evidence to support negotiations on drug costs to a certain degree. In order to enhance the selection of more efficacious first-line drugs, it is imperative to have enhanced prognostic markers and precise risk stratification of patients.

This study offers several noteworthy advantages. Firstly, based on the most up-to-date evidence, this study represents one of the initial cost-effectiveness analysis directly comparing serplulimab and placebo with chemotherapy among ESCC patients. Many researchers acknowledge the potential of immune checkpoint inhibitors (ICIs) as promising interventions for ESCC patients. These inhibitors are gaining approval from the NMPA for the first-line ESCC intervention. Nonetheless, limited attention has been given to value-focused economic evaluations of serplulimab. Secondly, we employed a Markov model to simulate clinical and financial consequences, facilitating reproducibility by other researchers. Thirdly, the cost analysis of serplulimab considers the patient assistance programs (PAP) provided by the China Primary Health Care Foundation, aligning the results with the Chinese health system. Fourthly, we performed a subgroup analysis to evaluate the economic outcomes for the six subgroups defined in the ASTRUM-007 trial, providing valuable subgroup-specific information for treatment decision-making. Finally, we present a thorough cost-effectiveness discussion that considers the variances in healthcare environments across various areas of China, providing insightful information for medical professionals, governments, and financial organizations.

Our model has several limitations stemming from simplifying disease processes, costs, and model assumptions. Firstly, similar to others, our simulation model relied on clinical trial data and is thus inherently susceptible to uncertainty. However, the Weibull models firmly adhered to the survival data, and their accuracy was confirmed using sensitivity analysis. Future updates from the ASTRUM-007 trial are required to clarify the ambiguity regarding the long-term benefits of serplulimab and chemotherapy in treating aESCC. Secondly, this analysis did not account for the costs associated with grade 1 or 2 adverse events (AEs), potentially impacting the study findings. However, the sensitivity analysis demonstrated that the results remained unchanged when the AE-related parameters were assorted within the prespecified range. Thirdly, the selection of utility values assumes a crucial role in pharmacoeconomic analyses. Utility scores from the ASTRUM-007 experiment were not available. Hence the numbers we used in our analyses were from advanced EC-reported values. Results of univariate sensitivity studies showed that PFS and PD utility values were the primary factors influencing model outcomes. However, tornado charts illustrated that the ICERs remained below the predefined threshold, irrespective of the variations in these values within the predetermined range.

This study provides objective data reference for clinicians’ clinical decision-making, patient individualized treatment, national health insurance decision-making and guideline updating. Prior investigations have indicated that, as a first-line treatment for patients with esophageal squamous cell carcinoma (ESCC), serplulimab lacks cost-effectiveness compared to chemotherapy. However, upon thorough scrutiny, it was observed that several of these studies took into account drug donations or favorable company policies, thus not entirely reflecting real-world research outcomes.

## Conclusion

In summary, based on the economic evaluation conducted using our decision analytical model, our study demonstrates that serplulimab plus chemotherapy is a highly productive intervention for aESCC patients who are positive for PD-L1 relative to chemotherapy only, considering a WTP cutoff of 3 folds of the GDP per capita within the Chinese healthcare system context. The significant improvement in OS and PFS observed with the combination of serplulimab and chemotherapy underscores the importance of engaging in talks and discussions about the pricing of serplulimab. This is essential to address the economic consequences associated with its application effectively. These efforts are necessary to ensure the approval of appropriate treatment guidelines. Moreover, the results of our study hold promise for augmenting the importance attributed by patients and healthcare professionals to the early identification of biomarkers. This, in turn, could facilitate a more individualized and refined approach to optimizing cancer treatment strategies.

### Electronic supplementary material

Below is the link to the electronic supplementary material.

**Supplementary Material 1: Figure S1.** Model structure. **Figure S2.** Kaplan-Meier curve fitting and extrapolation. **Figure S3.** Probability sensitivity analysis scatter plot. **Table S1.** CHEERS checklist. **Table S2.** Drug dose and cost. **Table S3.** Summary of statistical goodness-of-fit of K-M curve. **Table S4.** Subgroup analysis results

## Data Availability

All authors had unrestricted access to the study data and assumed full accountability for its integrity and accuracy. The datasets produced and examined in this study are available upon reasonable request from the corresponding author.
