# Supplementary material for: A cost-effectiveness analysis of the combination of serplulimab with chemotherapy for advanced esophageal squamous cell carcinoma: insights from the ASTRUM-007 trial
Source: Cost Eff Resour Alloc. 2024 Jan 27;22:8. doi: 10.1186/s12962-024-00516-5 (PMC10821310; doi:10.1186/s12962-024-00516-5)
Supplement: Supplementary file 1 — Supplementary Material 1: Figure S1. Model structure. Figure S2. Kaplan-Meier curve fitting and extrapolation. Figure S3. Probability sensitivity analysis scatter plot. Table S1. CHEERS checklist. Table S2. Drug dose and cost. Table S3. Summary of statistical goodness-of-fit of K-M curve. Table S4. Subgroup analysis results [file 12962_2024_516_MOESM1_ESM.docx]

**Figure S1.** Model Structure.

**Figure S2.** Kaplan-Meier Curve Fitting and Extrapolation.

**Figure S3.** Probability Sensitivity Analysis Scatter Plot.

**Table S1.** CHEERS Checklist.

**Table S2.** Drug Dose and Cost.

**Table S3.** Summary of Statistical Goodness-of-fit of K-M Curve.

**Table S4.** Subgroup Analysis Results.

**Figure S1. Model Structure.**


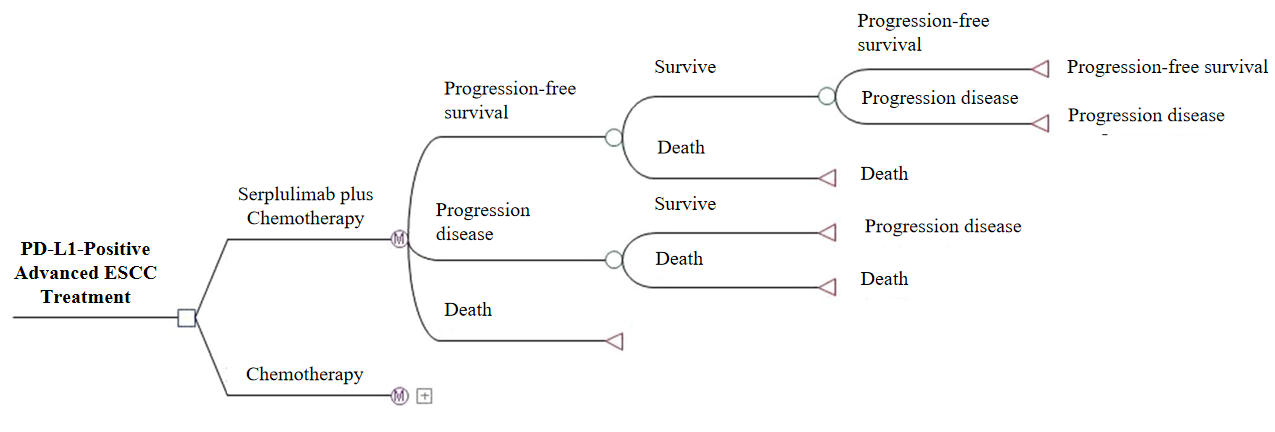


Abbreviation: PD-L1, programmed death ligand 1; CF, cisplatin and 5-fluorouracil; M, Markov.

**Figure S2. Kaplan-Meier Curve Fitting and Extrapolation.**

**
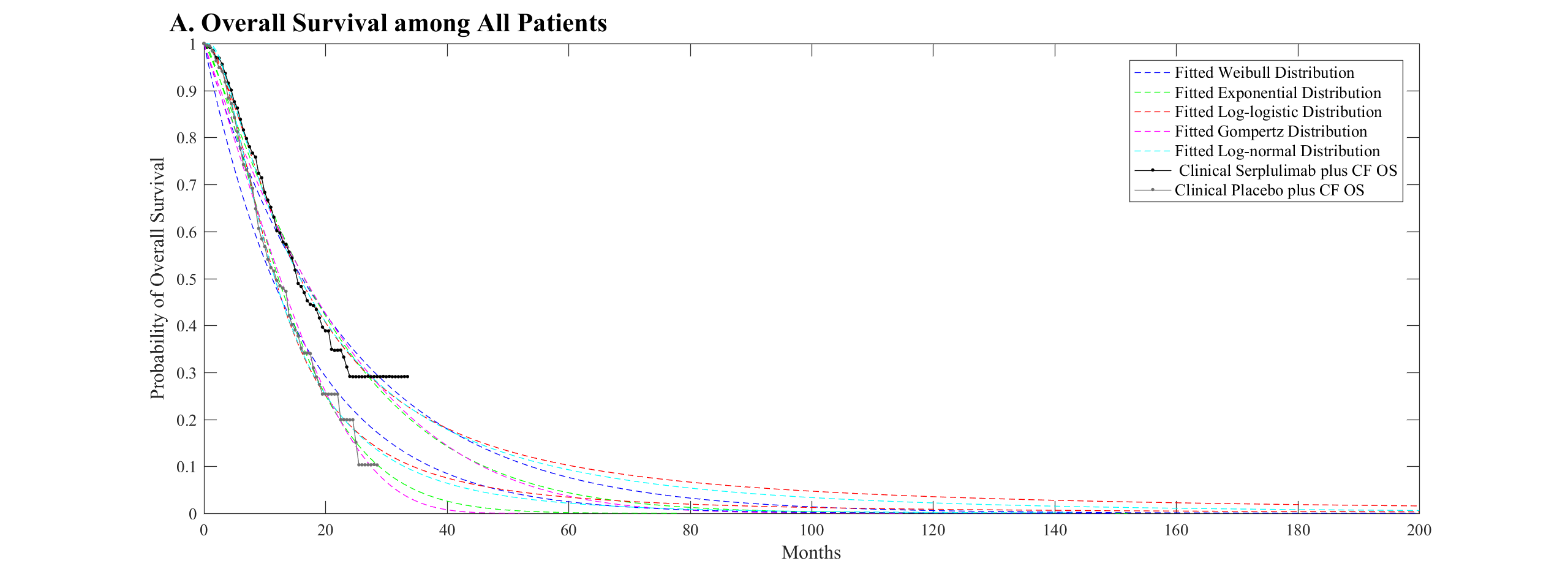
**

**
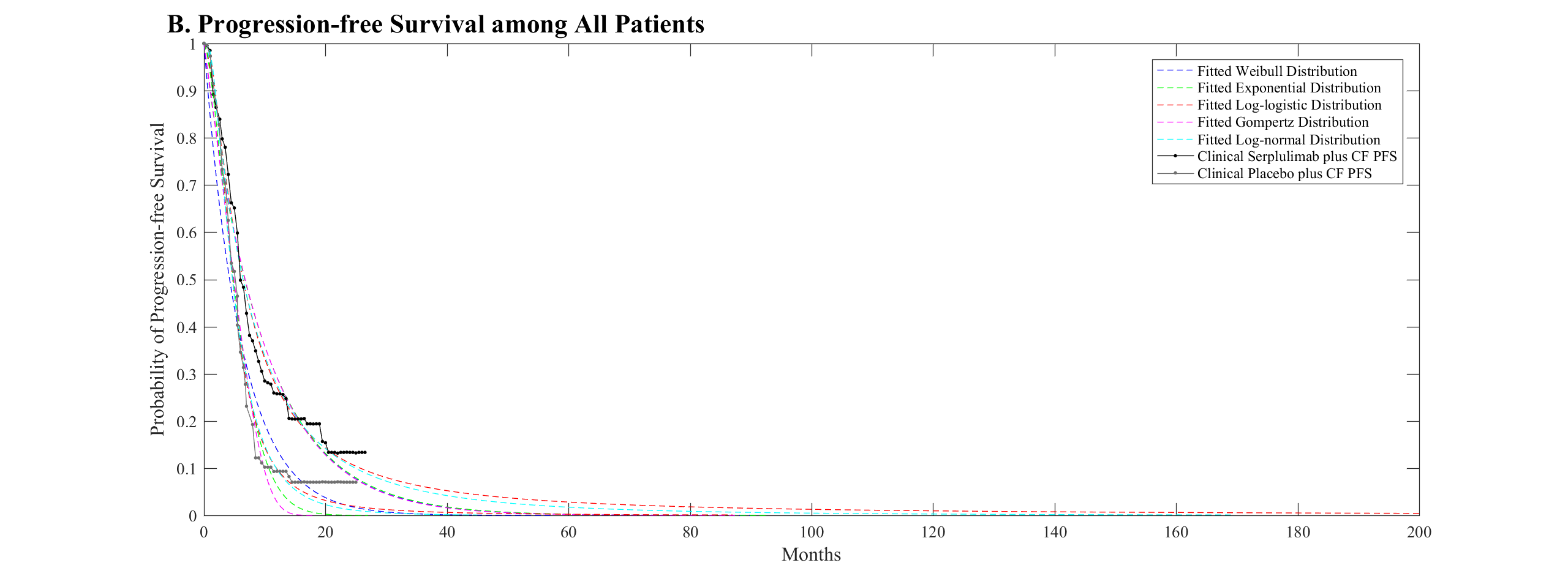
**

**
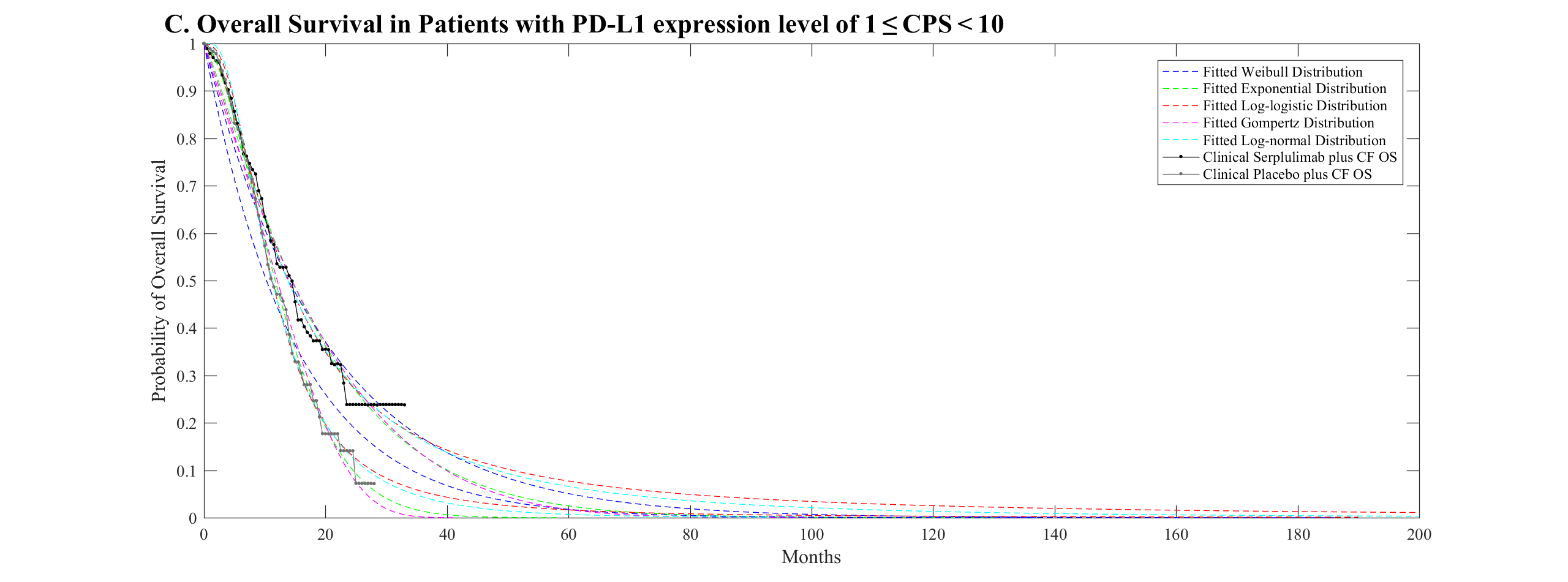
**

**
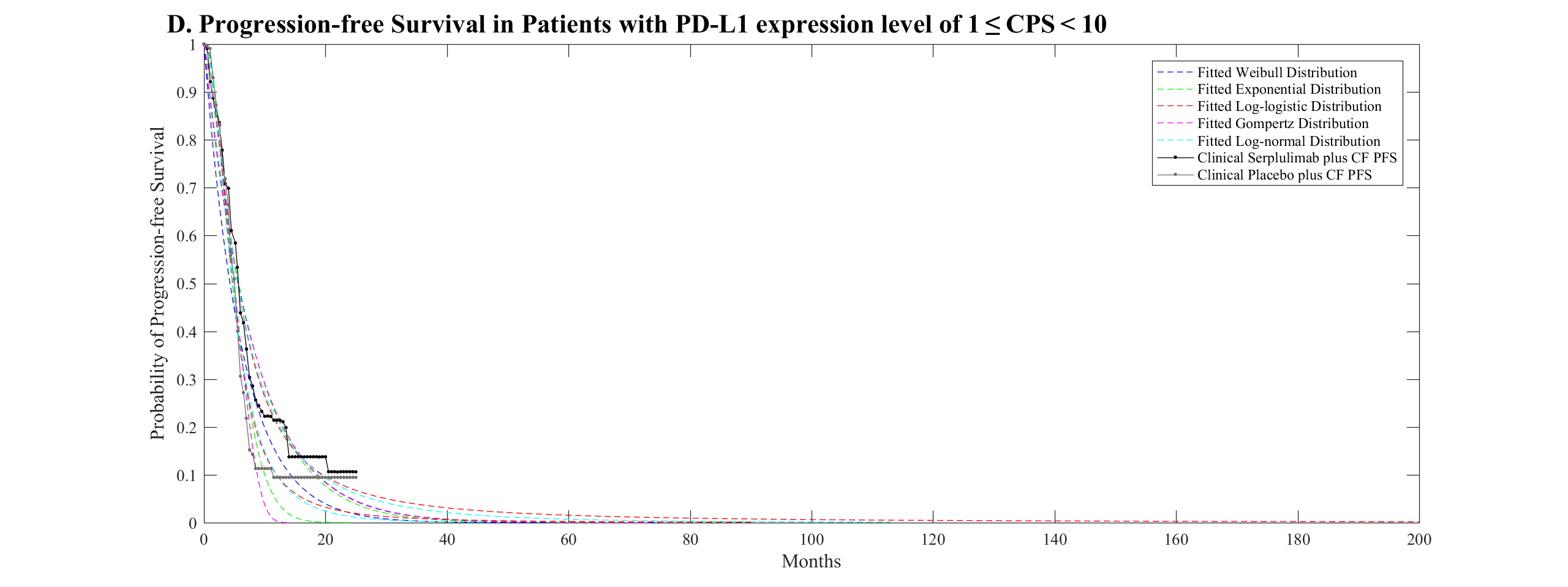
**

**
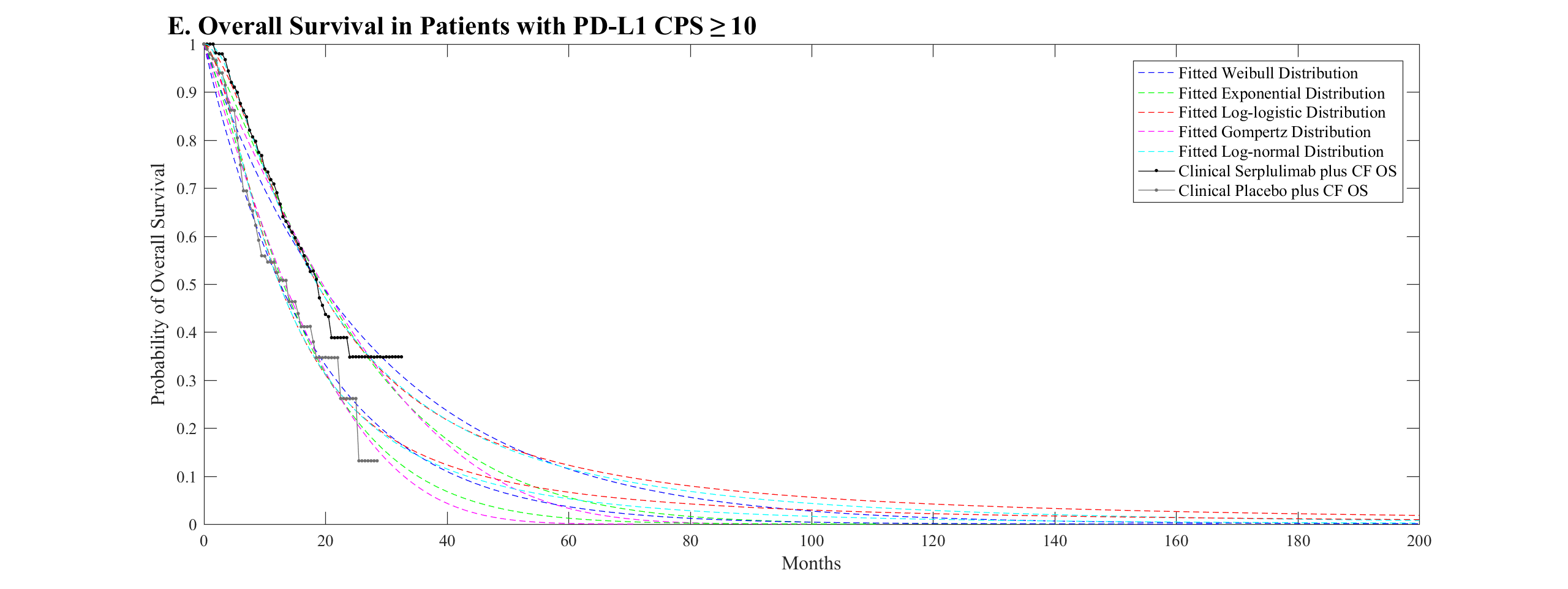
**

**
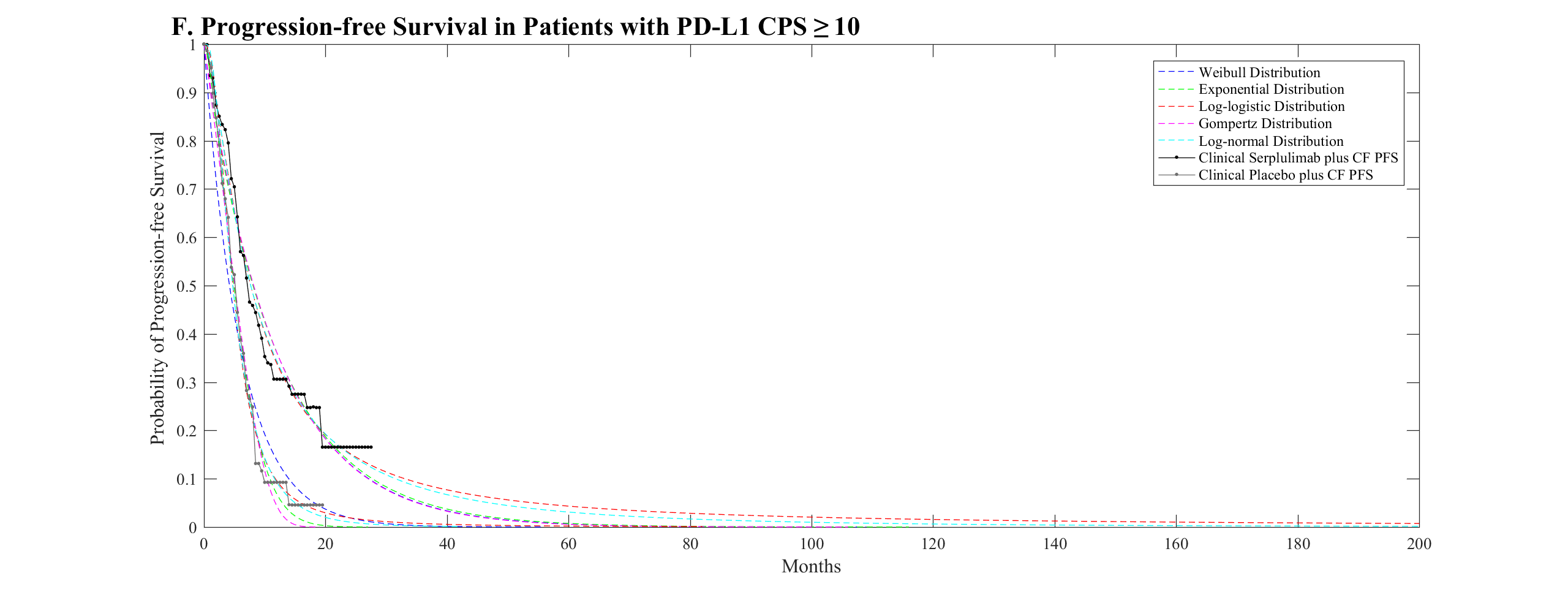
**

Abbreviation: OS, overall survival; PFS, progression-free survival; CF, cisplatin and 5-fluorouracil; PD-L1, programmed death ligand 1; CPS, combined positive score.

Kaplan-meier curve fitting and extrapolation for overall survival in the overall patients (A), progression-free survival in the overall patients (B), overall survival in patients with PD-L1 expression level of 1 ≤ CPS < 10 (C), progression-free survival in patients with PD-L1 CPS ≥ 10 (D), overall survival in patients with PD-L1 expression level of 1 ≤ CPS < 10 (E), progression-free survival in patients with PD-L1 CPS ≥ 10 (F), respectively.

**Figure S3.** **Probability Sensitivity Analysis Scatter Plot.**


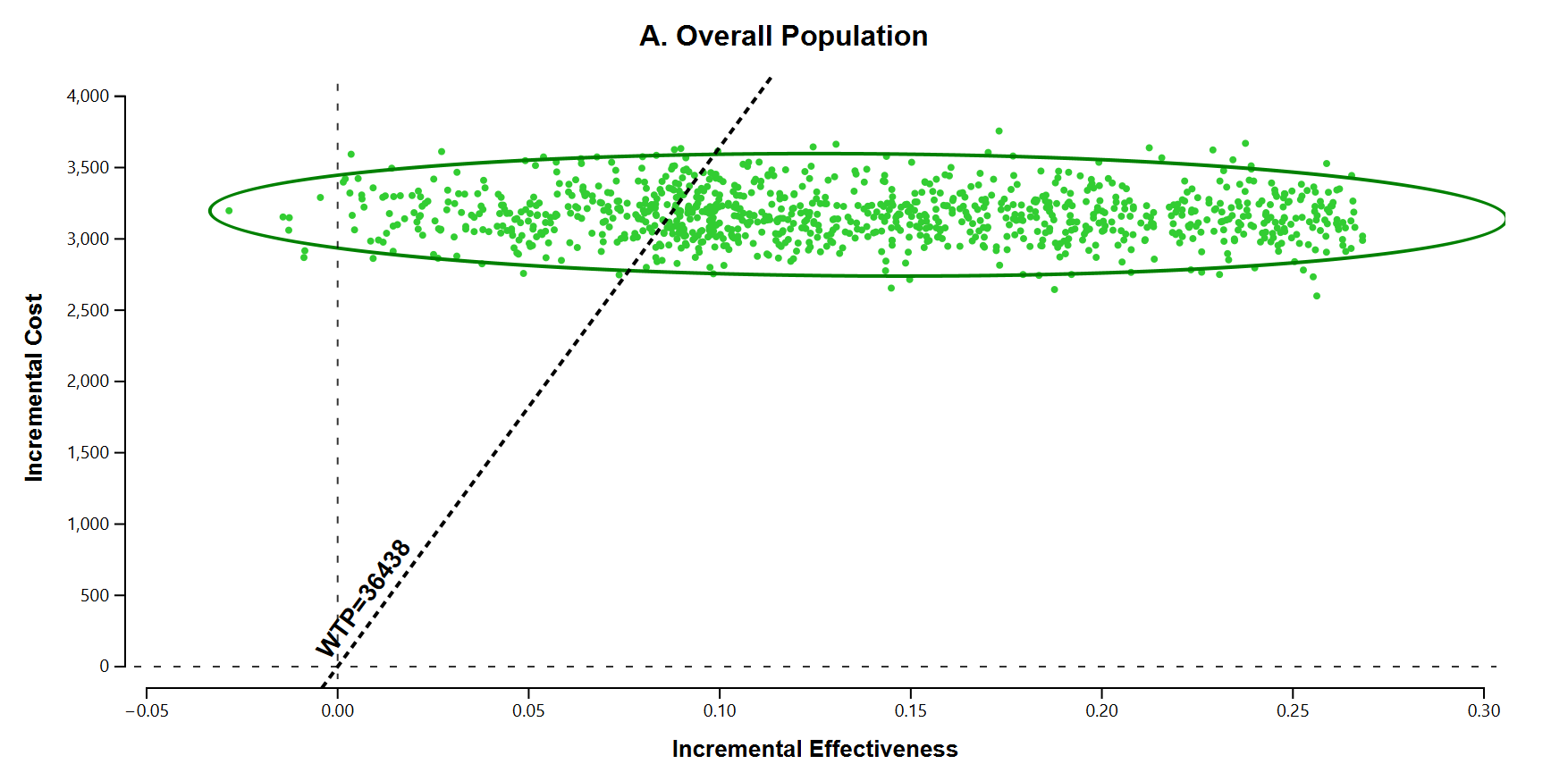


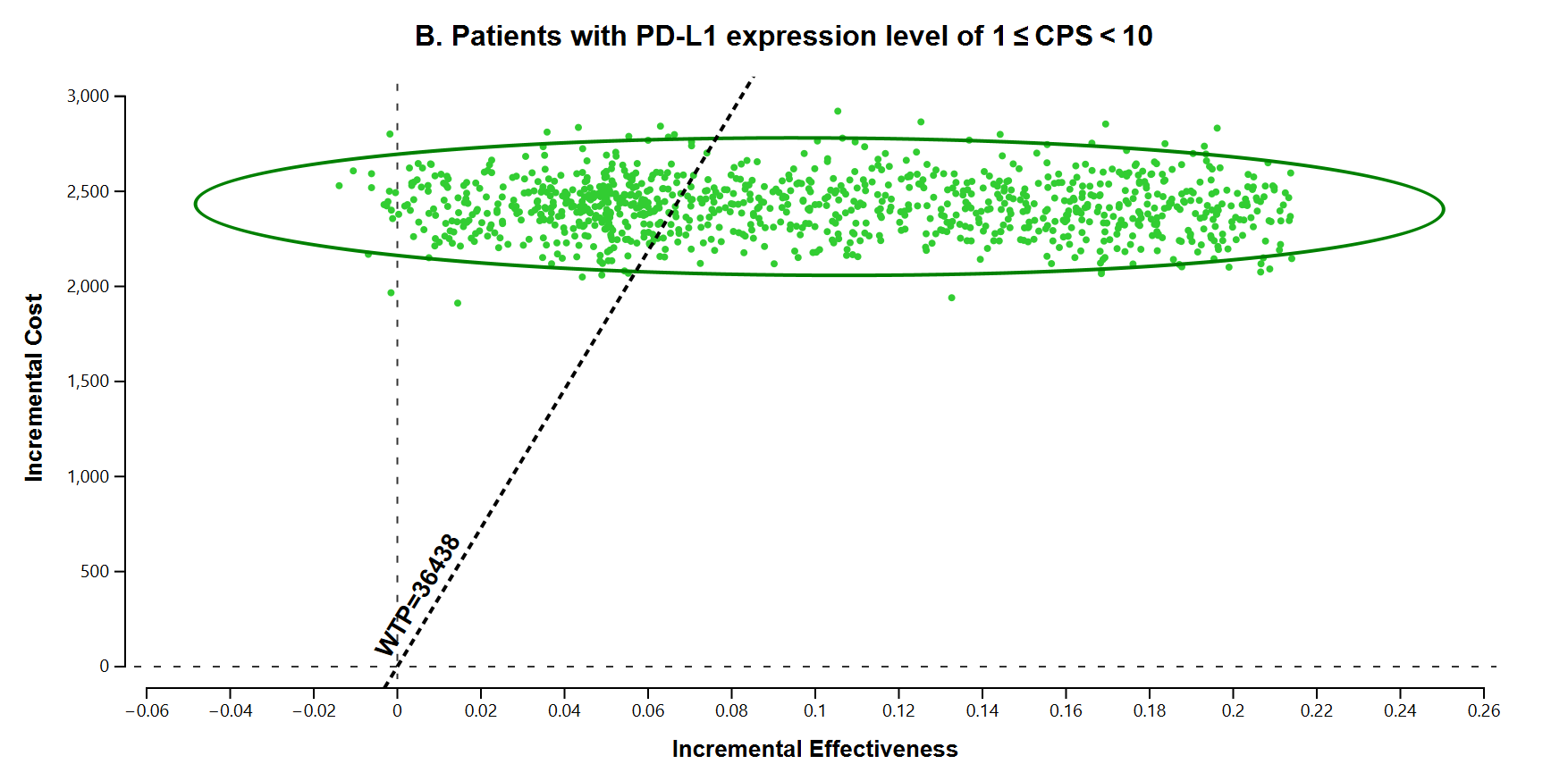


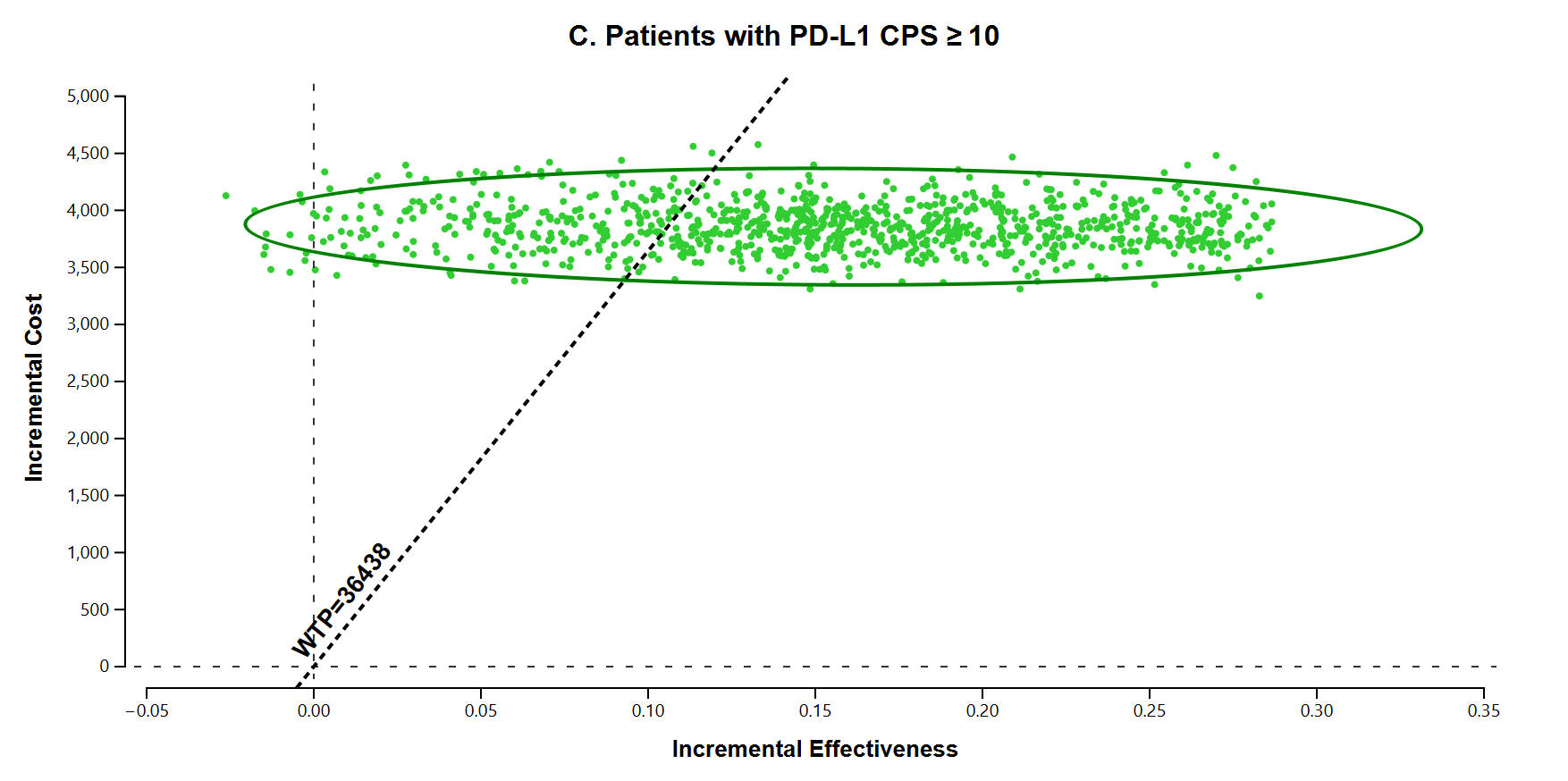
Abbreviation: CF, cisplatin and 5-fluorouracil; WTP, willingness-to-pay.

Each point in the diagram represents a simulation result of 10,000 Monte Carlo simulations. The ellipse represents the 95% CI and the dotted line represents WTP of $36,438/QALY in the overall patients (A), patients with PD-L1 expression level of 1 ≤ CPS < 10 (B), patients with PD-L1 CPS ≥ 10 (C).

**Table S1. CHEERS Checklist.**

| **Section/item** | **Item No** | **Recommendation** | **Reported?** |
| --- | --- | --- | --- |
| **Title and abstract** | | | |
| Title | 1 | Identify the study as an economic evaluation or use more specific terms such as “cost-effectiveness analysis”, and describe the interventions compared. | Y |
| Abstract | 2 | Provide a structured summary of objectives, perspective, setting, methods (including study design and inputs), results (including base case and uncertainty analyses), and conclusions. | Y |
| **Introduction** | | | |
| Background and objectives | 3 | Provide an explicit statement of the broader context for the study. | Y |
|  |  | Present the study question and its relevance for health policy or practice decisions. |  |
| **Methods** | | | |
| Target population and subgroups | 4 | Describe characteristics of the base case population and subgroups analysed, including why they were chosen. | Y |
| Setting and location | 5 | State relevant aspects of the system(s) in which the decision(s) need(s) to be made. | Y |
| Study perspective | 6 | Describe the perspective of the study and relate this to the costs being evaluated. | Y |
| Comparators | 7 | Describe the interventions or strategies being compared and state why they were chosen. | Y |
| Time horizon | 8 | State the time horizon(s) over which costs and consequences are being evaluated and say why appropriate. | Y |
| Discount rate | 9 | Report the choice of discount rate(s) used for costs and outcomes and say why appropriate. | Y |
| Choice of health outcomes | 10 | Describe what outcomes were used as the measure(s) of benefit in the evaluation and their relevance for the type of analysis performed. | Y |
| Measurement of effectiveness | 11a | *Single study-based estimates:*Describe fully the design features of the single effectiveness study and why the single study was a sufficient source of clinical effectiveness data. | Y |
|  | 11b | *Synthesis-based estimates:*Describe fully the methods used for identification of included studies and synthesis of clinical effectiveness data. |  |
| Measurement and valuation of preference based outcomes | 12 | If applicable, describe the population and methods used to elicit preferences for outcomes. | Y |
| Estimating resources and costs | 13a | *Single study-based economic evaluation:*Describe approaches used to estimate resource use associated with the alternative interventions. Describe primary or secondary research methods for valuing each resource item in terms of its unit cost. Describe any adjustments made to approximate to opportunity costs. | Y |
|  | 13b | *Model-based economic evaluation:*Describe approaches and data sources used to estimate resource use associated with model health states. Describe primary or secondary research methods for valuing each resource item in terms of its unit cost. Describe any adjustments made to approximate to opportunity costs. |  |
| Currency, price date, and conversion | 14 | Report the dates of the estimated resource quantities and unit costs. Describe methods for adjusting estimated unit costs to the year of reported costs if necessary. Describe methods for converting costs into a common currency base and the exchange rate. | Y |
| Choice of model | 15 | Describe and give reasons for the specific type of decision-analytical model used. Providing a figure to show model structure is strongly recommended. | Y |
| Assumptions | 16 | Describe all structural or other assumptions underpinning the decision-analytical model. | Y |
| Analytical methods | 17 | Describe all analytical methods supporting the evaluation. This could include methods for dealing with skewed, missing, or censored data; extrapolation methods; methods for pooling data; approaches to validate or make adjustments (such as half cycle corrections) to a model; and methods for handling population heterogeneity and uncertainty. | Y |
| **Results** | | | |
| Study parameters | 18 | Report the values, ranges, references, and, if used, probability distributions for all parameters. Report reasons or sources for distributions used to represent uncertainty where appropriate. Providing a table to show the input values is strongly recommended. | Y |
| Incremental costs and outcomes | 19 | For each intervention, report mean values for the main categories of estimated costs and outcomes of interest, as well as mean differences between the comparator groups. If applicable, report incremental cost-effectiveness ratios. | Y |
| Characterising uncertainty | 20a | *Single study-based economic evaluation:*Describe the effects of sampling uncertainty for the estimated incremental cost and incremental effectiveness parameters, together with the impact of methodological assumptions (such as discount rate, study perspective). | Y |
|  | 20b | *Model-based economic evaluation:*Describe the effects on the results of uncertainty for all input parameters, and uncertainty related to the structure of the model and assumptions. |  |
| Characterising heterogeneity | 21 | If applicable, report differences in costs, outcomes, or cost-effectiveness that can be explained by variations between subgroups of patients with different baseline characteristics or other observed variability in effects that are not reducible by more information. | Not  applicable |
| **Discussion** | | | |
| Study findings, limitations, generalisability, and current knowledge | 22 | Summarise key study findings and describe how they support the conclusions reached. Discuss limitations and the generalisability of the findings and how the findings fit with current knowledge. | Y |
| **Other** | | | |
| Source of funding | 23 | Describe how the study was funded and the role of the funder in the identification, design, conduct, and reporting of the analysis. Describe other non-monetary sources of support. | Y |
| Conflicts of interest | 24 | Describe any potential for conflict of interest of study contributors in accordance with journal policy. In the absence of a journal policy, we recommend authors comply with International Committee of Medical Journal Editors recommendations. | Y |

A good template page for CHEERS Checklist is as follows: <http://www.ispor.org/TaskForces/EconomicPubGuidelines.asp.>

Reference:

*Husereau D, Drummond M, Petrou S, et al. Consolidated health economic evaluation reporting standards (CHEERS)—Explanation and elaboration: A report of the ISPOR health economic evaluations publication guidelines good reporting practices task force. Value Health 2013;16:231-50.*

**Table S2. Drug Dose and Cost.**

| **Drug** | **Dose** | **Time** | **Unit costs ($/mg)** |
| --- | --- | --- | --- |
| Serplulimab plus CF | Serplulimab, 3mg/kg | Administered intravenously every 2 weeks for a total duration of up to 2 years. | 0.6873 |
|  | Cisplatin, 50 mg/m^2^ | Administered intravenously every 2 weeks for up to 8 cycles | 0.1432 |
|  | 5-fluorouracil, 1,200 mg/m^2^ | Administered intravenously every 2 weeks for up to 12 cycles | 0.0320 |
| Placebo plus CF | Cisplatin, 50 mg/m^2^ | Administered intravenously every 2 weeks for up to 8 cycles | 0.1432 |
|  | 5-fluorouracil, 1,200 mg/m^2^ | Administered intravenously every 2 weeks for up to 12 cycles | 0.0320 |
| Paclitaxel | Paclitaxel, 250 mg/m^2^ | Administered intravenously every 3 weeks | 1.1220 |

Abbreviation: CF, cisplatin and 5-fluorouracil.

**Table S3. Summary of Statistical Goodness-of-fit of K-M Curve.**

|  | **Exponential** | **Weibull** | **Gompertz** | **Log-logistic** | **Log-normal** |
| --- | --- | --- | --- | --- | --- |
| **Overall patients** | | | | | |
| **Serplulimab plus CF OS curve** | | | | | |
| AIC | 103.4661 | **99.6842** | 103.0082 | 102.6036 | 102.2183 |
| BIC | 107.9051 | **101.9037** | 107.4472 | 107.0426 | 106.6573 |
| **Placebo plus CF OS curve** | | | | |  |
| AIC | 113.8524 | **104.0150** | 115.5058 | 110.2550 | 110.4387 |
| BIC | 117.9732 | **106.0755** | 119.6267 | 114.3759 | 114.5596 |
| **Serplulimab plus CF PFS curve** | | | | | |
| AIC | 149.7511 | **143.6623** | 150.9497 | 145.4482 | 145.6446 |
| BIC | 153.729 | **146.6513** | 154.9276 | 149.4262 | 149.6226 |
| **Placebo plus CF PFS curve** | | | | | |
| AIC | 347.7231 | **212.1932** | 860.3354 | 227.2144 | 241.3695 |
| BIC | 351.5868 | **214.1251** | 864.1990 | 231.0781 | 245.2331 |
| **Patients with PD-L1 expression level of 1 ≤ CPS < 10** | | | | | |
| **Serplulimab plus CF OS curve** | | | | | |
| AIC | 116.7402 | **111.7325** | 116.1301 | 115.1520 | 114.8495 |
| BIC | 121.1496 | **113.9372** | 120.5395 | 119.5614 | 119.2589 |
| **Placebo plus CF OS curve** | | | | | |
| AIC | 128.5366 | **109.4774** | 134.1008 | 121.3347 | 122.0116 |
| BIC | 132.6227 | **111.5204** | 138.1869 | 125.4208 | 126.0977 |
| **Serplulimab plus CF PFS curve** | | | | | |
| AIC | 165.7877 | **145.6410** | 162.0372 | 157.0253 | 157.9613 |
| BIC | 169.6513 | **146.5729** | 165.9009 | 160.8890 | 161.8250 |
| **Placebo plus CF PFS curve** | | | | | |
| AIC | 409.7710 | **208.2670** | Inf | 225.3110 | 238.1551 |
| BIC | 413.6346 | **210.1988** | Inf | 229.1746 | 242.0187 |
| **Patients with PD-L1 CPS ≥ 10** | | | | | |
| **Serplulimab plus CF OS curve** | | | | | |
| AIC | 82.4544 | **79.3893** | 82.4636 | 81.9602 | 81.6000 |
| BIC | 86.8337 | **81.5790** | 86.8429 | 86.3395 | 85.9793 |
| **Placebo plus CF OS curve** | | | | | |
| AIC | 97.7078 | **93.3458** | 98.2932 | 96.0541 | 96.0560 |
| BIC | 101.8287 | **95.4062** | 102.4141 | 100.1750 | 100.1769 |
| **Serplulimab plus CF PFS curve** | | | | | |
| AIC | 133.6548 | **116.7542** | 134.9269 | 131.1376 | 131.0782 |
| BIC | 137.7055 | **117.7795** | 138.9776 | 135.1883 | 135.1289 |
| **Placebo plus CF** **PFS curve** | | | | | |
| AIC | 183.9926 | **130.7029** | 262.5848 | 145.9453 | 151.5167 |
| BIC | 187.3704 | **132.3918** | 265.9626 | 149.3230 | 154.8944 |

Abbreviation: CF, cisplatin and 5-fluorouracil; OS, overall survival; PFS, progression-free survival; AIC, Akaike’s information criterion; BIC, Bayesian information criterion; PD-L1, programmed death ligand 1; CPS, combined positive score; Inf, infinite.

The AIC and BIC value of the distributions and the visual fitting of the curves (Figure S2) showed that the Weibull distribution is probably the most reasonable parametric survival model. Weibull distribution is flexible and widely used for matching patients with three states over time because it can monotonously increase or decrease risk functions, and it is suitable for estimating events occurring during early follow-up work.

**Table S4. Subgroup Analysis Results.**

| **Subgroups** | **HR of OS**  **(95% CI)** | **HR of PFS**  **(95% CI)** | **ICER,**  **$/QALY** | **Probability of**  **cost-effectiveness, %** |
| --- | --- | --- | --- | --- |
| **Age, years** |  |  |  |  |
| ＜65 | 0.62 (0.45-0.87) | 0.61 (0.45-0.83) | 25,409 | 69.02 |
| ≥65 | 0.76 (0.52-1.12) | 0.57 (0.41-0.81) | 36,277 | 57.92 |
| **Sex** |  |  |  |  |
| Male | 0.67 (0.51-0.88) | 0.59 (0.46-0.75) | 28,605 | 68.14 |
| Female^a^ | 0.47 (0.21-1.03) | 0.41 (0.19-0.85) | NA | NA |
| **ECOG performance status** |  |  |  |  |
| 0 | 0.43 (0.24-0.78) | 0.49 (0.30-0.81) | 19,706 | 77.34 |
| 1 | 0.70 (0.53-0.93) | 0.65 (0.50-0.85) | 29,723 | 62.27 |
| **Disease status** |  |  |  |  |
| Locally advanced | 0.52 (0.26-1.04) | 0.71 (0.35-1.44) | 19,890 | 66.92 |
| Distantly metastatic | 0.70 (0.54-0.92) | 0.58 (0.46-0.74) | 30,847 | 65.22 |
| **Smoking status** |  |  |  |  |
| Current or former | 0.65 (0.47-0.89) | 0.55 (0.42-0.74) | 28,013 | 70.12 |
| Never | 0.73 (0.47-1.14) | 0.64 (0.43-0.97) | 32,299 | 59.47 |

^a^ HR unavailable due to the small sample size in this subgroup.

Abbreviation: HR, hazard ratio; OS, overall survival; PFS, progression-free survival; CI, confidence interval; QALYs, quality-adjusted life-years; ICER, incremental cost-effectiveness ratio; INMB, incremental net monetary benefit; INHB, incremental net health benefits; NA, not available; ECGO, Eastern Cooperative Oncology Group.
